# A new species of *Phymatodes* Mulsant (Coleoptera, Cerambycidae) from China

**DOI:** 10.3897/zookeys.367.5774

**Published:** 2014-01-06

**Authors:** Shulin Yang

**Affiliations:** 1 School of Life Sciences, Guizhou Normal University, Guiyang, Guizhou China 550001

**Keywords:** Guizhou, longhorn beetles, taxonomy

## Abstract

A new species *Phymatodes* (*Poecilium*) *latefasciatus* **sp. n.** (Coleoptera, Cerambycidae, Cerambycinae, Callidiini) from China is described and illustrated. Features distinguishing the new species from its congeners are presented.

## Introduction

*Phymatodes* Mulsant, 1839 is a genus in the tribe Callidiini Mulsant, 1839. Nearly 70 species of *Phymatodes* have been recorded around the world. There are 32 species described from the Palearctic region (Rapuzzi and Sama 2009, Löbl and Smetana 2010, Danilevsky 2010b, Sama et al. 2011). Gressitt (1951) recorded 10 *Phymatodes* species (actually 9 species) from China in subgenera, *Phymatodellus*, *Paraphymatodes* and *Poecilium*. The 10 species were *Phymatodes (Phymatodellus) kozlovi* Semenov & Plavilstshikov, 1936, *Phymatodes (Phymatodellus) semenovi* Plavilstshikov, 1935, *Phymatodes (Phymatodellus) sinensis* (Pic, 1900), *Phymatodes (Phymatodellus) ussuricus* Plavilstshikov, 1940, *Phymatodes (Paraphymatodes) albicinctus* (Bates, 1873), *Phymatodes (Paraphymatodes) hauseri* (Pic, 1907), *Phymatodes (Paraphymatodes) mediofasciatum* (Pic, 1933), *Phymatodes (Poecilium) infasciatus* Pic, 1935, *Phymatodes (Poecilium) maaki* (Kraatz, 1879), *Phymatodes (Poecilium) savioi* Pic, 1935. Afterwards, *Phymatodes (Phymatodellus) ussuricus* Plavilstshikov, 1940 was treated as a synonym of *Phymatodes (Phymatodellus) infasciatus* Pic, 1935 (Hua 1982), and four new species were described in the genus: *Phymatodes (Poecilium) mizunumai* Hayashi, 1974, *Phymatodes (Poecilium) eximium* Holzschuh, 1995, *Phymatodes (Phymatodellus) jiangi* Wang & Zheng, 2003, *Phymatodes (Phymatodellus) ahenum* Holzschuh, 2007. Totally 13 *Phymatodes* species are distributed in China.

Gressitt (1951) placed Chinese *Phymatodes* species in subgenus *Poecilium* by characters: elytra with a cluster of long erect hairs behind scutellum, reddish at base and blackish posteriorly, generally with two subobliquely transverse pale bands; eyes nearly divided by emargination. Ohbayashi and Niisato (2007) divided Japanese *Phymatodes* species into subgenus *Poecilium* by fewer characters: roughly equal length of first hind tarsal segment to total length of second and third segments; and presence of two elytral bands. Löbl and Smetana (2010) elevated the subgenus *Poecilium* to genus with subgenera *Paraphymatodes* and *Phymatoderus* as its synonyms. Later, *Poecilium* was re-placed as subgenus (Danilevsky 2010a). In this study, *Poecilium* is considered as a subgenus of *Phymatodes* by a synthesis of characters of Gressitt (1951) and Ohbayashi and Niisato (2007).

According to the above characters, currently, there are totally nine species in the subgenus *Poecilium*. Among the 13 Chinese *Phymatodes* species, *Phymatodes maaki* (Kraatz, 1879), *Phymatodes savioi* Pic, 1935, *Phymatodes mizunumai* Hayashi, 1974, and *Phymatodes eximium* Holzschuh, 1995 are recognized in subgenus *Poecilium*. Besides the four Chinese species, five other species in subgenus *Poecilium* were recorded: *Phymatodes alni* (Linné, 1767) (distribution: Europe; Asia: Kazakhstan, Turkey), *Phymatodes antonini* Rapuzzi, Sama & Tichy, 2011 (distribution: Syria), *Phymatodes ermolenkoi* (Tsherepanov, 1980) (distribution: Russia: Primorskii Krai Province), *Phymatodes kasnaki* Sama, 2011 (distribution: Turkey), and *Phymatodes quadrimaculatum* Gressitt, 1935 (distribution: Japan).

Recently, specimens representing a new species of *Phymatodes* from Guizhou province of China were discovered and it fits into the subgenus *Poecilium* according to aforementioned characters. The new species is described herein.

The collection acronyms used in the text are as follows:

GZNULS School of Life Sciences, Guizhou Normal University, Guiyang, Guizhou China.

## Taxonomy

### Phymatodes (Poecilium) latefasciatus sp. n.

http://zoobank.org/DB67D24A-8EAC-4613-861B-48E72B23D6A0

http://species-id.net/wiki/Phymatodes_latefasciatus

Figure 1

#### Etymology.

The name refers to the second elytral band which is gradually widening toward suture after mid-point.

#### Diagnosis.

Characters of the new species conform with all established characters of subgenus *Poecilium*, elytra with a cluster of long erect hairs behind scutellum, reddish at base and blackish posteriorly, with two pale bands; eyes nearly divided by emargination; ratio of first hind tarsal segment’s length to total length of second and third segments nearly 1:1 (Gressitt 1951, Ohbayashi and Niisato 2007). Thus, it is placed in the subgenus *Poecilium*.

**Figure 1.**
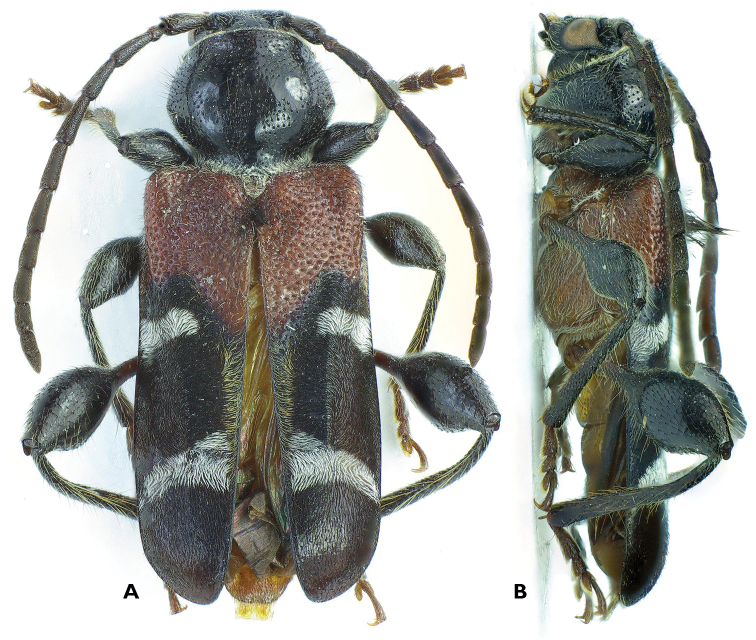
*Phymatodes (Poecilium) latefasciatus*, sp. n. **A** dorsal view **B** lateral view.

*Phymatodes (Poecilium) latefasciatus* sp. n. is distinguished from its congeners by the combination of the following characters: shape of the second elytral band; proportion of reddish brown area on basal elytron to the whole elytron; the color of antennae; and shapes of swelling part of femur. *Phymatodes latefasciatus* can be distinguished from all other congeners by the shape of its 2^nd^ elytral band which is gradually widening towards suture after mid-point (Fig. 1a). In addition to this most obvious one, other characters distinguish the new species from three other morphologically close Chinese species in the same subgenus: *Phymatodes (Poecilium) maaki* (Kraatz, 1879) (distribution: China: Heilongjiang, Jiangxi, Sichuan, Taiwan; Russia: Far East; Korea, Japan) (Kraatz 1879, Hua 2002, Löbl and Smetana 2010); *Phymatodes (Poecilium) savioi* Pic, 1935 (distribution: China: Jiangxi, Shanghai) (Pic 1935, Hua 2002, Löbl and Smetana 2010); and *Phymatodes (Poecilium) mizunumai* Hayashi, 1974 (distribution: Taiwan) (Hayashi 1974, Hua 2002, Chou 2008, Löbl and Smetana 2010). The new species is also different from *Phymatodes maaki* (Kraatz, 1879), a species most similar to the new species, by these characters: 1) proportion of the size of reddish brown area on elytral base to the whole elytron smaller, approximately 1/3; 2) antenna black; 3) clubbed part of meso-femora and hind femora shorter comparing to length of whole femur, suddenly swelling toward tibiae after middle. Beside shape of second elytral band, *Phymatodes latefasciatus* also differs from *Phymatodes savioi* Pic, 1935 by characters: 1) smaller proportion of reddish brown basal elytral area; 2) antenna black; 3) elytral bands ivory not yellowish, and first band not reaching suture. As the above two species, three other characters are also used to distinguish *Phymatodes latefasciatus* from *Phymatodes mizunumai* Hayashi, 1974, 1) antenna black; 2) first elytral band nearly transverse, not arcuate and shape as a caret symbol; 3) ratio of femoral clubbed part to femoral basal part larger.

#### Description.

Female (male unknown). Moderate body size, length 8.4–8.5 mm (holotype 8.4 mm), width 2.9–3.2 mm (measured across humeri, holotype 2.9 mm). Yellowish-brown, head, prothorax, swollen part of femurs black, antennae and tibiae lighter, basal part of elytra (ca 1/3 of total elytral length) reddish brown, the rest black, two subobliquely transverse ivory bands on each elytron.

Front nearly flat, transverse; head slightly concave between antennal tubercles which are slightly raised and separated by approximately the width of one antennal socket, front and vertex with sparse punctures and short yellow pubescences on some of these punctures; eyes coarsely-faceted; scape and antennomere 2 with semi-erect long yellow hairs, rest of the antennomeres covered with short dark brown hairs, antennomeres 2, 3 and 4 with sparse long yellow hairs especially at tips, relatively long yellow hairs also presenting at ends of antennomeres 5 to 10 but length gradually reduced by antenna segment; outer tips of antennomeres 6 to 10 slightly serrated.

Prothorax transverse, approximately 1.2 times wide as long, widest and slightly angulated laterally near middle, contracting towards base and narrowest at base; pronotum slightly convex, shining, with irregular punctures and covered with moderately long erect black setae; short thick pale yellow hairs at collar edge and base of pronotum, forming a narrow transverse strip at the base; prosternal intercoxal process short, spine shaped, not reaching coxal middle; scutellum reddish yellow, length longer than width, nearly rectangle, semicircular at end.

Elytron long, approximately 4 times as basal width, parallel sided, with rounded apex; basal third red brown area irregularly dense punctured, with erect hairs, a cluster of long erect brown hairs after scutellum; rest of the elytron covered with dense black hairs except two nearly transverse ivory bands and a narrow golden strip lengthwisely along suture between the two bands; the first ivory band extending from elytral margin, approximately half of elytron width, not reaching suture; the second ivory band slightly curving towards base, gradually widening towards suture after mid-point of elytron, nearly reaching suture but interrupted by the lengthwisely golden strip.

Femora strongly swollen, fore-femur swollen gradually from near fore-coxa, meso-femur and hind femur swollen after approximately half of femoral length; legs cover with moderately long thick pale golden hairs, hair on tibiae longer and darker than that on femora; non-swollen part of femora reddish brown, swollen parts black; 1^st^ segment of hind tarsi short, approximately 1.2 times as long as 2^nd^ and 3^rd^ together.

Abdomen slightly shining with small punctures, with relatively sparse moderately long semi-erect pale yellow hairs.

#### Type material.

Holotype ♀ from Heichong, Shibing County, Guizhou Province, CHINA, 2013.IV.17, 27°09.068'N, 108°07.384'E, net sweeping on a *Viburnum* sp. plant, S. Yang col. (**GZNULS**). Paratype ♀ from Leigongshan, Leishan County, Guizhou Province, CHINA, 26°22.781'N, 108°11.534'E, 2012.V.23, Lindgren funnel trap, S. Yang col. (**GZNULS**).

##### Modified couplets to key to Chinese *Phymatodes* species of subgenus *Poecilium*

A modified key to *Phymatodes* species of subgenus *Poecilium* is presented based on Gressitt’s (1951, page 228) key to Chinese *Phymatodes* species. In his key, *Phymatodes latefasciatus* will run to couplet 8. Couplets 8–9 can be modified, as presented blow, to accommodate the new species and other species described in the subgenus after his key published.

**Table:** 

8	Dark portions of elytra with two silvery white bands; first band subtransverse, not reaching suture	9
–	Dark portions of elytra with two yellowish gray bands; first band arched; reaching suture; second band transverse; only hind femoral clubs pitchy	*Phymatodes savioi* Pic
9	Second elytral band subtransverse, reaching or nearly reaching suture, not caret symbol shaped	10
–	Second elytral band oblique, not reaching suture, caret symbol shaped; front wrinkled with ridges	*Phymatodes eximium* Holzschuh
10	Width of whole second elytral band roughly constant, not gradually widening towards suture	11
–	Width of whole second elytral band not constant, gradually widening towards suture after middle	*Phymatodes latefascitus* Yang
11	First elytral band arcuate and acute angled as a caret symbol; ratio of femoral clubbed part to un-swelling femoral basal part smaller	*Phymatodes mizunumai* Hayashi
–	First elytral band slightly bend smoothly, not acute angled as a caret symbol; ratio of femoral clubbed part to un-swelling femoral basal part larger	*Phymatodes maaki* Kraatz

## Supplementary Material

XML Treatment for Phymatodes (Poecilium) latefasciatus
